# A Review of Potential Use of Amazonian Oils in the Synthesis of Organogels for Cosmetic Application

**DOI:** 10.3390/molecules27092733

**Published:** 2022-04-24

**Authors:** Luis Eduardo Mosquera Narvaez, Lindalva Maria de Meneses Costa Ferreira, Suellen Sanches, Desireé Alesa Gyles, José Otávio Carréra Silva-Júnior, Roseane Maria Ribeiro Costa

**Affiliations:** 1 Laboratory of Pharmaceutical Nanotechnology, College of Pharmacy, Federal University of Pará, Belém 66075-110, Brazil; lemosqueran@unal.edu.co (L.E.M.N.); lindalva.costa.ferreira@ics.ufpa.br (L.M.d.M.C.F.); suellen.sanches@yahoo.com.br (S.S.); 2 Jamaica College of Health Sciences, School of Pharmacy, University of Technology, 237 Old Hope Road, Kinston 6, Jamaica; desiree.gyles@gmail.com; 3 Laboratory of Pharmaceutical and Cosmetic R&D, College of Pharmacy, Federal University of Pará, Belém 66075-110, Brazil; carrera@ufpa.be

**Keywords:** transport, vegetable oil, organogelator, skin, oxidative stability, minority polar components

## Abstract

New strategies for the delivery of bioactives in the deeper layers of the skin have been studied in recent years, using mainly natural ingredients. Among the strategies are organogels as a promising tool to load bioactives with different physicochemical characteristics, using vegetable oils. Studies have shown satisfactory skin permeation, good physicochemical stability mainly due to its three-dimensional structure, and controlled release using vegetable oils and low-molecular-weight organogelators. Within the universe of natural ingredients, vegetable oils, especially those from the Amazon, have a series of benefits and characteristics that make them unique compared to conventional oils. Several studies have shown that the use of Amazonian oils brings a series of benefits to the skin, among which are an emollient, moisturizing, and nourishing effect. This work shows a compilation of the main Amazonian oils and their nutraceutical and physicochemical characteristics together with the minority polar components, related to health benefits, and their possible effects on the synthesis of organogels for cosmetic purposes.

## 1. Introduction

**Figure 1:**
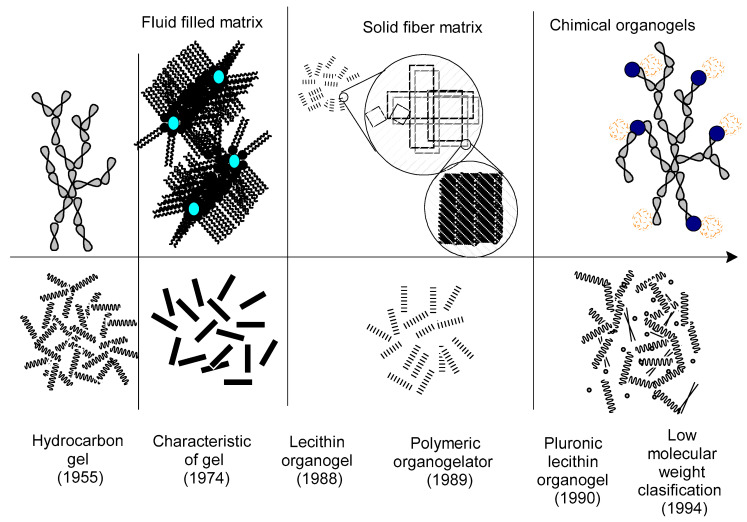
For a long time, gels have evolved in their way of synthesis, especially in the cosmetic area, where the organogels of Lecithin promoted research into the synthesis of new organogels based on polymeric organogelators and low molecular weight. The latter, by its versatility, low cost of synthesis, and high physicochemical stability, is ideal for permeation of bioactives in the deepest layers of the skin.

Amazonian natural oils are widely used in food [1], cosmetics [2,3], and medicine [4,5] due to their physical-chemical characteristics that depend on the extraction method, as already reported in the works of Dong et al. [6], Martin et al. [7], and Divine et al. [8]. Oil extraction methods include conventional (mechanics and use of solvents) and non-conventional (supercritical fluid extraction, ultrasound, microwave, and enzyme-assisted extraction), the latter not yet implemented industries due to their high operating costs and low yields, with the mechanical extraction being the most used in the industry for being free of solvents within the conventional ones [8,9,10]. This method used in these Amazonian species ensures the presence of predominantly polyunsaturated fatty acids and exceptional nutraceutical substances, which are not found in traditional natural oils [1,11,12]. There is a huge variety of applications of Amazonian oils mainly in the cosmetic area; therefore, the demand for research to develop innovative applications and formulate new products rises daily. The main objective of this review was to provide detailed information on the physicochemical characteristics and the therapeutic and nutraceutical properties of Amazonian oils, as well as their potential in applications as a vehicle for bioactive molecules, mainly in the formulation of organogels for dermal application, as a sustainable, economical, and efficient alternative. Recently, there have been many investigations focused on the use of these oils for cosmetic formulations such as microemulsions [13], nanoemulsions [14,15], and solid lipid nanoparticles [16], among others. This article provides a context for a discussion on the variety of Amazonian oils eligible for organogel synthesis and their potential application as controlled release systems for bioactive molecules. The synthesis method was reviewed, from the perspective of the solvent (natural oil) and the gelling agent to identify the physicochemical properties of the organogel. Figure 1 shows the important milestones of the organogel. This article analyzed the use of traditional natural oils in the synthesis of the organogel, going from the selection of the organogelator to the characterization of the used oils and their effect on the oxidative stability and degree of skin rewards. In order to propose the use of Amazonian oils in the synthesis of organogels that can be transformed into differently solvents due to their own characteristics, transporting bioactive molecules, and can even generate synergism with the molecules of some oils which have already been identified with biological activity. Such as limonoids from andiroba (insect repellent, anti-malaria), sesquiterpenes from copaiba (antibacterial and anti-inflammatory) and B-carotenoids from buriti (antioxidants and photoprotective), among others.

## 2. Methodology

Studies with original data related to the proposed topic (published between 1992 and 2020) were identified by searching electronic databases and reviewing citations. Among the databases were Elsevier, ScIELO, web of Science, and Springer, including publications in English, Spanish, and Portuguese. Eligible studies for this review included in vivo studies in experimental animals or cell cultures, with prospective, parallel, or crossover designs, with full text, whose results were positive. Articles whose main information was not related to Amazon organogels and oils were excluded.

## 3. Organogel

Gels are structural elements that contain a three-dimensional network formed mainly by physical interactions that involve hydrogen bond, electrostatic interactions, Van der Waals forces, and hydrophobic forces, among others [17,18,19]. These molecular bonds are affected by the type of solute and solvent (natural oil) used in the formulation as well as the pH, temperature, agitation, cooling, and ionic strength; so, they can be designed according to their desired application, modifying each variable described above [20]. Organogels can be defined as lipophilic solid and liquid combinations in which solid–lipid structure materials (organogelators) are incorporated in low concentrations and with the proper processing (heating, agitation, and cooling, for example), are dispersed in the oil phase, and self-assembled, trapping the liquid oil to form 3-D networks of the structuring material in the oil [21]. 

Organogels can be classified by the size of their oil structuring molecules into high or low molecular weight. High-molecular-weight organogelators (HMOGs) are mainly polymers, structured by an organic solvent forming physical or chemical interactions between strands, resulting in a supramolecular network. Low-molecular-weight organogelators (LMOGs) can be further subdivided by the oil structuring method, through the formation of a three-dimensional network of crystalline particles or a fibrillar network that fills space [19]. 

LMOGs are frequently used in cosmetology for their desirable properties of physical organization within the oil phase and their ability to gel organic solvents in small amounts. Low-molecular-weight organogelators have a number of advantages in the application of organogels. The solid fibrillar matrix of the organogels improves the mechanical properties, resulting in a better encapsulation rate of lipophilic or amphiphilic molecules, with a homogeneous distribution within the particles’ structural framework. The active ingredient, which can be in liquid state, is able to be directly gelled and keep itself as the main component of the formulation; the consistency can be easily controlled depending on the concentration of the organogelator [22,23,24]. The majority of physical organogels are prepared by heating the mixture of the solid and the liquid component that forms the organic solution/dispersion, called the first phase. The cooling of the mixture follows, which fixes the formation of the gel, known as the second phase. In the second phase, the solubility of the structural agent in the liquid phase decreases and the organogelator–solvent interactions are reduced, resulting in the molecules of the structural agent “coming out” of solution [22,25]. The synthesis of physical organogels occurs by means of the organogelator solubilization chosen in a concentration of 0.1% to 15% in the heated solvent, depending on the nature of the structural agent [26,27,28]. 

**Figure 2:**
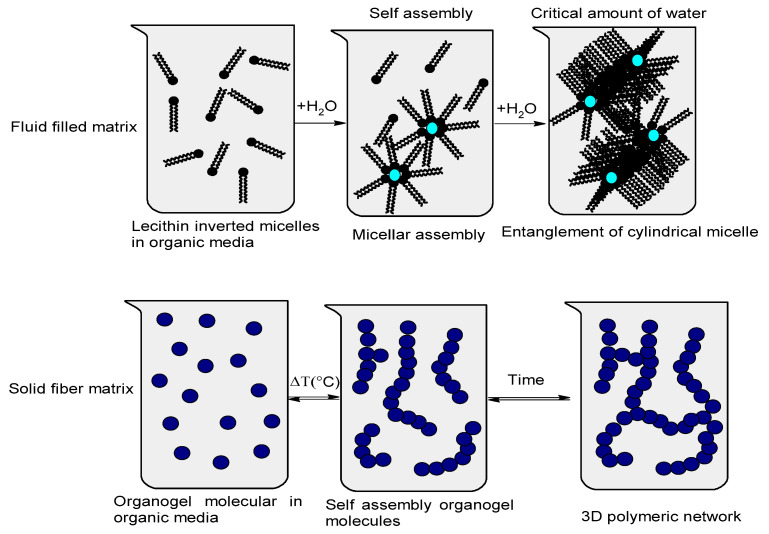
Preparation methods that influence organogel structures. **Top panel**: example of a liquid-filled matrix. Amphiphilic lecithin molecules organize into inverted micelles in an organic solvent. **Bottom panel**: example of a solid fiber matrix. The dissolution of organogelator molecules in an organic solvent at high temperature leads to a concentrated solution [32].

As the temperature decreases, the affinity between the organogelator and the solvent molecules decreases. The organogel’s molecules begin to self-assemble to form solid aggregates trapped in a three-dimensional network with an increased intermolecular physical interaction. On the other hand, fluid-filled organogel matrices structure the organic solvents in the same way as the solid fiber matrix (Figure 2): The size of the aggregates increases and the eventual entanglement of these structures immobilizes the solvent as a result of surface tension. They are thermoreversible systems and can be transparent or opaque. The critical difference arises in the kinetic behavior of the two types of matrices. While solid matrices have a robust and permanent morphology during the useful life of the gel, fluid matrices are transient structures in constant dynamic remodeling [29]. 

### 3.1. The Selection of the Organogel

In recent times, a wide variety of organogelators has been investigated, making it necessary to establish criteria for their use, especially in the cosmetic and pharmaceutical industries. In this sense, the following should be considered: (1) the processes and conditions involved in organogel synthesis, such as temperature, solute–solvent interaction, and the gelling concentration, (2) biosafety, and (3) effectiveness. These are relevant in order to achieve a safe design and effective pharmaceutical and cosmetic chargers. 

Within the process of organogel synthesis, conditions, such as temperature, in which the structuring material dissolves in the vegetable oil, followed by the solute–solvent interactions, are variables that affect a specific gelling system. However, the organogelator concentration and the gelling temperature affect the topological and the microstructure of the gel network. All this is achieved mainly through various degrees of supersaturation in the initial stage of the gelling process [30,31]. Depending on the organogelator, the temperature can range between 40 °C to 90 °C, depending on the melting point and affinity of this for the vegetable oil.

Furthermore, there are reported in literature organogels synthesized at high temperatures. For example, in the investigation of Toro-Vazquez et al. [33], using candelilla wax and vegetable oil as organogelators, a temperature of 90 °C was needed for their synthesis, while, in the works of Rocha et al. [34] and ÖgÜtcü et al. [35], using candelilla wax with soybean oil and beeswax with extra virgin olive oil, respectively, they achieved a temperature of 80 °C. Other researchers reported temperatures of up to 140 °C [36] to solubilize carnauba wax in canola oil and beeswax in grapeseed oil.

For that reason, this type of organogelator with a high melting point has an effect on the quality of the oil, since heating at a high temperature produces changes in the fatty acid constituents of the triglyceride molecules. These result in the alteration of the physicochemical properties of the vegetable oil due to the change in chain length and degree and position of unsaturation [37,38]. Some studies reported that the thermal heating of sunflower oil to 99 ± 2 °C caused a high level of deterioration by losing a significant amount of essential fatty acids (linoleic acid) with the development of oxidative rancidity [39], which is how these and other thermolabile constituents of the oil are altered.

The thermolabile actives loaded in the molecular network of the organogel would also be strongly affected, which is why structuring materials with a high melting point such as phytosterols, ceramides, fatty acids, fatty alcohols, long-chain monoglycerides and diglycerides, and waxes is not convenient when using polyunsaturated oils and thermolabile actives. Waxes especially present a variety of contradictory results in terms of critical gelation concentrations, organogel texture, and oxidative stability, among others, since it is believed that some minor components in waxes can significantly affect the physical properties of organogels [40] and can act as pro-oxidants [35,36,41]. Other factors that can cause variation in the quality of the organogel from waxes are the harvest season, the manufacturing conditions, and the purity.

Solvent quality is one way to describe and predict solvent–gelling interactions. Using the theory of Hansen’s solubility parameter (HSP) [42], the quality of the solvent can be quantified and subsequently adjusted, so that the gelling agent has an optimal solubility in oil [43]. This phenomenon is widely studied for the formation of organogels. In the work of Gravelle [41], the Hansen solubility parameter was used to modify the oily phase by adding mineral oil and castor oil to soybean oil, resulting in gels with different mechanical properties [19]. Additionally, two fundamental parameters to take into account when selecting organogelators are safety and efficiency. Biocompatibility encompasses a wide spectrum of biological characteristics that can only be defined by analyzing the various forms of interaction of the organism with the material, with special emphasis on the tissue–material interface [44]. The use of biocompatible, biodegradable, and non-immunogenic organogelators makes them safe for long-term application. They also have greater therapeutic efficacy and decrease fluctuating related to the release of active compounds [45,46].

According to the American National Standard Institute (ANSI), there are levels for the evaluation of biocompatibility, among which, initially, it is necessary to apply in vitro tests, using cell cultures [47]. Fibroblasts and keratinocytes are the main cellular components of the dermal and epidermal layers, respectively, that can be cultured to investigate biocompatibility [48]. The first criterion to verify the biocompatibility of a material is to evaluate its cytotoxic potential [48]. Many of the low-molecular-weight organogelators for dermatological and cosmetic purposes have already been proven to be biocompatible and safe [49]. Similarly, it has been proven that the use of biocompatible oils, such as soybean, sunflower, grapeseed, or mustard oils, can reduce problems of irritability, allergy, or immunological reactions caused by other types of organic solvents [32,50].

### 3.2. General Considerations of Lipids (Natural Oils)

Lipids are defined as small hydrophobic or amphipathic molecules that can be formed wholly or partially by condensation reactions of carbanion-based ketoacyl thioesters and/or by condensation of carbocation-based isoprene units. Taking into account this classification system, lipids have been divided into eight categories: fatty acids, glycerolipids, glycerophospholipids, sphingolipids, saccharolipids, polyketides (derived from the condensation of ketoacyl subunits), sterol lipids, and prenol lipids (derived from the condensation of isoprene subunits) [51,52].

The building blocks that constitute vegetable oils are mainly triglycerides (triacylglycerol TGA), a triple alcohol glycerol structure linked to three fatty acids (FA) by means of ester linkages. With less monoacylglycerides (MAG) and diacylglycerols (DAG), saturated, unsaturated, and polyunsaturated fatty acids such as linoleic and linolenic acid can also be found together with fat-soluble vitamins (A, D, E, and K) [19,20,53]. The latter provides multiple benefits to the skin such as rehydration, elimination of free radicals generated by UV radiation, and acceleration of healing processes (re-epithelialization). In addition, fatty acids in general are natural emollients that provide vitality to the skin. Vegetable oils as well as their constituents can be considered as triglyceride solvents, whose polarities are determined by the ratio of saturated/unsaturated fatty acids, the length of the fatty acid chain, the conformation of the triglyceride chain, and the presence of acids’ polar/unsaturated fats [19,20,54]. 

To facilitate the study of lipids, chemists have divided them into two main classes: the unsaponifiable ones (terpenes, steroids, and eicosanoids), which are normally less than 2%, and the saponifiable, which in turn are divided into simple lipids and complex lipids [52,55,56]. Simple lipids are those that are easy to hydrolyze to simpler constituents. They are those that produce, at most, two types of products by hydrolysis. Most simple lipids are esters of long-chain carboxylic acids called fatty acids. The two main groups of fatty acid esters are waxes and glycerides. Waxes are long-chain alcohol esters and glycerides are glycerin esters [56,57]. Acylglycerols are partially soluble in fat and water, and, for this reason, MAG and DAG are mainly used as surfactants [58].

In the synthesis of organogels, it has been shown that oils rich in long-chain monounsaturated fatty acids and polyunsaturated fatty acids promote glycerol self-assembly monostearate in polymorphic networks, mainly of the β consonance crystalline phase [59]. These authors also observed that the degree of unsaturation, polarity, and length of the fatty acid chains influence the stability of these self-assembled lipids [58]. Complex lipids are those that are not easily hydrolyzed in an acidic or basic aqueous solution. Within this group are glycerophospholipids, glycosphingolipids, and glycoglycerolipids, among others, giving three or more products by hydrolysis [51]. These compounds have amphipathic characteristics that could affect the synthesis and characteristics of organogels depending on the organogelator used in their manufacturing.

## 4. Influences of Solvent Polarity on the Formation of Organogel

The oil selection in organogelation processes has a significant impact on the physicochemical characteristics and texture of the organogel network, either due to the amount of saturated, unsaturated, or polyunsaturated materials present, in addition to their minor constituents. Among the minority polar components are emulsifiers, degradation products (oxidation, hydrolysis, thermal degradation), and natural compounds, such as tocopherols, phytosterols, water, free fatty acids, and mono-diglycerides [60].

An investigation carried out by Scharfe et al. [61] proposed that the minority of polar components in the oil interact with the organogelator, resulting in harder gels at lower concentrations of polar compounds. At higher concentrations, a negative impact was observed on the resistance of the gel due to saturation in interactions with the organogelator. Elasticity and oil loss are examples of other gel properties that can be altered by modifying the polarity of the oil [31]. In the work of Imai [62], the ability of solid paraffin wax (C32) to gel oils of different polarities was explored. The more polar oil resulted in a harder gel, in which a rougher platelet wax crystal lattice was formed, caused by disorder of the lamellar structure at the nanometer scale. In this way, changing the polarity of the oils can alter the structure and mechanical resistance of the corresponding organogels. The appearance of firmer gels is also attributed to oils rich in highly unsaturated fatty acids that have a greater degree of freedom for the conformation of the lipid chain [63]. The properties of the gel were analyzed as a function of the permittivity of the oil, which reflected the concentration of minor polar compounds, as well as the quality of the oil.

The important impact of solvent polarity on the gelling of natural organogels was confirmed by Dassanayake et al. [64], where the structuring material interacted with olive oil, camellia oil, and salad oil (canola oil:soybean oil = 1:1) at a minimum gelling concentration of 1.0% by weight. The viscosity of the salad oil was the lowest compared to the other two oils; in contrast, the hardness at a concentration of 10% of the structuring material was lower for the salad oil compared to the other oils. The type of oil should, therefore, have some kind of effect, as there is a higher oleic acid concentration and decreased linoleic acid for olive and camellia oil when compared to salad oil [19].

In addition to the texture and the viscosity, the morphology of the crystals is altered depending on the type of solvent. Beeswax crystals revealed variable morphologies in different oils: Large spherulite crystals were observed in canola, soybean, and sunflower oils, and needle-shaped crystals were observed in corn, olive, and safflower oils [65]. It is not yet clear what the mechanism of these compounds in the gelling process is or what their detailed impact on the properties of organogels is, as more studies are necessary to clarify: Their effect, possible interactions, mechanism of action, and critical concentrations in which the texture can vary. For this reason, the appropriate selection of the organogelator and the oil has a specific relationship to the formulation and development of cosmetic and pharmaceutical products [40].

In the special case of fluid-filled matrices, organogels such as lecithin use a polar agent as a third component, which acts as a stabilizing and structure-forming agent. A number of polar solvents have been studied for their suitability to produce the thickening effect in solutions of hydrocarbons and fatty acid esters of lecithin. Water has been widely used as a polar solvent for organogel formation. However, it has been established that glycerol, formamide, and ethylene glycol have the ability to induce gelling [66]. 

The ability to promote gelling of lecithin solutions has been correlated with the polarity of the solvent used and its physicochemical properties [66,67]. This correlation is particularly pronounced in the series of structurally related solvents such as glycerol and ethylene glycol. In proposed models of organogels, solvent molecules form bridges between the phosphate groups of neighboring lipid molecules, allowing their association in tubular aggregates through an extensive network of hydrogen bonds. The organic solvent plays a vital role in the organogels by providing the desired solvent action for the drug (hydrophobic) as well as for the lecithin. Natural oils including soybean oil, sunflower oil, grapeseed oil, and mustard oil are proposed as potentially useful organic solvents for preparing lecithin organogels [66].

## 5. Amazonian Oil and Their Use in the Formation of Organogels

The Amazon region is rich with oleaginous plants. The rich oils from these species are largely uninvestigated. Natural Amazonian oils produced by these species have a unique composition, in addition to their physicochemical (Table 1) and nutraceutical properties (Table 2) and some polar minority compounds (Table 3) [1]. The research for new sources of vegetable oils has been of great interest in recent decades; not only for the food industry [68], but in the cosmetic industry, oils are used as humectants, emollients, emulsifiers, and viscosity adjusters [69]. Myristic, palmitic, stearic, linoleic, and linolenic acids are very common in certain types of cosmetics such as soaps and shampoos, while other fatty acids have rejuvenating or healing properties [70]. 

The nutraceutical properties of natural Amazonian oils are not limited only to their lipid composition, but also include the presence of other substances, called unsaponifiable matter, which has important biological properties [68]. Bioactive substances that can be found in vegetable oils include some fat-soluble vitamins, which have a protective action against the evolution of the natural degenerative processes that lead to diseases and premature aging; as examples, vitamins E (tocopherols) and β-carotene (provitamin A) are present in several species of oil [68]. These antioxidant substances are extremely important to reduce the harmful effects of free radicals generated by oxidative processes in our metabolism [71].

Of the species that deserve to be highlighted for their high oil content are acai (*Euterpe oleracea* Mart.), andiroba (*Carapa guianensis* Aubl.), babassu (*Attalea speciosa* Mart.), buriti (*Mauritia flexuosa* L.f.), copaiba (*Copaifera officinalis* L.), cumaru (*Dipteryx odorata* Aubl.), inchi (*Caryodendron orinocense* H. Karst), patawa (*Oenocarpus bataua* Mart.), pequi (*Caryocar brasiliense* Cambess.), pracaxi (*Pentaclethra macroloba* Willd.), sacha inchi (*Plukenetia volubilis* L.), and tucumã (*Astrocaryum vulgare* Mart.).

**Table 1:** Physicochemical properties of different Amazonian vegetable oil by alphabetical order; (-): not reported.

Vegetal Oil	Appearance (25 °C)	Color	Smell	Acidity Index	Peroxide Index	Iodine Value	Saponificatio Index	Refractive Index	Density	Unsaponified Material (Bioactive)	Fusion Point	Reference
Acai	Líquid	Green	Chara	1.20–1.60 mg KOH/g	1.26 meq H_2_O_2_/kg	70 g I_2_/100 g	175.69 mg KOH/g	1.481 (40 °C)	0.893 g/mL	2–3%	-	[72,73]
Andiroba	Líquid	Yellow to brown	Chara	3.89 mg NaOH/g	1.96 meq O_2_/kg	89.77 g I_2_/100 g	232.84 mg KOH/g	1.4611 (40 °C)	0.98 g/mL	3–5%	22 °C	[74]
Babassu	Solid	-	Chara	3.47 mg KOH/g	2.40 meq O_2_/kg	14.0 g I_2_/100 g	265 mg KOH/g	1.451 (40 °C)	0.9280 g/mL	0.40%	22–26 °C	[75]
Buriti	Liquid	Red	Chara	3.12 mg NaOH/g	14.12 meq O_2_/kg	74.64 g I_2_/100 g	192.88 mg KOH/g	1.4610 (40 °C)	0.909 g/mL	0.5%	-	[76]
Cumaru	Liquid	Green	Chara	0.22 mg KOH/g	<10 meq H_2_O_2_ kg	67 g I_2_/100 g	212.3 mg KOH/g	1.460 (40 °C)	0.935 g/mL	4.9%	69–73 °C	[73]
Inchi	Liquid	Translucent yellow	Chara	3 g NaOH/g	7.16 meq O_2_/kg	136.53 g I_2_/100 g	176.93 mg KOH/g	1.4734 (25 °C)	0.9065 g/mL	1.0%	−14.33 °C	[77]
Patawa	Liquid	Green	Chara	2 mg NaOH/g	<10.0 meq O_2_/kg	84 g I_2_/100 g	192 mg KOH/g	1.468 (40 °C)	0.9140 g/mL	1.30%	16 °C	[78]
Pequi	Liquid	Yellow	Chara	5.4 g NaOH/g	7.94 meq O_2_/kg	50 g I_2_/100 g	206.8 mg KOH/g	-	-	-	-	[79]
Pracaxi	Liquid	Translucent yellow	Chara	3 mg NaOH/g	5 meq O_2_/kg	68 g I_2_/100 g	170–180 mg KOH/g	1.461 (40 °C)	0.9173 g/mL	<1.5%	18.5 °C	[73]
Sacha inchi	Liquid	Translucent yellow	Chara	0.10 mg NaOH/g	2.77 meq O_2_/kg	189.16 g I_2_/100 g	189.60 mg KOH/g	14.816 (20 °C)	0.9255 g/mL	-	-	[80]
Tucuma	Liquid	Green	Chara	5.47 mg NaOH/g	2.99 meq O_2_/kg	12.7 g I_2_/100 g	202.71 mg KOH/g	1.461 (40 °C)	0.9100 g/mL	<1.8%	27 °C	[81,82]

**Table 2:** Fatty acid profile of different Amazonian vegetable oils by alphabetical order; (-): not reported.

	Acai	Andiroba	Babassu	Buriti	Cumaru	Inchi	Patawa	Pequi	Pracaxi	Sacha Inchi	Tucumã
Acids	Composition										
Caprylic Acid (C 8: 0)	-	-	6.21%	-	-	-	-	-	-	-	1.94%
Capric Acid (C 10: 0)	-	-	5.78%	-	-	-	-	-	-	-	0.80%
Lauric acid (C 12: 0)	0.07%	-	47.40%	0.03%	-	-	1.37%	-	1.20%	-	-
Miristic acid (C 14: 0)	0.13%	-	15.64%	0.08%	-	0.1%	0.94%	0.36%	0.71%	-	-
Palmitic acid (C 16: 0)	21.78%	31.40%	8.01%	16.78%	6.70%	10.3%	11.04%	29.48%	1.95%	6.30%	22.99%
Palmitoleic acid (C 16: 1)	3.26%	0.26%	0.02%	0.32%	-	0.1%	0.41%	0.66%	-	-	-
Margaric Acid (C 17: 0)	-	-	0.02%	0.08%	-	0.2%	-	-	-	-	-
Stearic acid (C 18: 0)	2.17%	10%	3.15%	1.77%	4.53%	3.4%	5.09%	2.44%	2.92%	3.81%	2.95%
Oleic acid (C 18: 1-Omega 9)	57.42%	50.6%	11.28%	74.06%	53.37%	11.8%	74.18%	59.99%	47.57%	10.45%	67.62%
Linoleic acid (C 18: 2-Omega 6)	11.08%	5.4%	1.85%	4.94%	16.45%	85.6%	5.97%	6.44%	12.08%	36.80%	1.15%
Linolenic acid (C 18: 3-Omega 3)	0.59%	-	0.25%	1.04%	3.32%	-	0.51%	-	1.07%	50.41%	4.97%
Arachidonic acid (C 20: 4)	-	-	-	-	0.70%	-	0.5%	-	1.34%	-	-
Arachidic acid (C 20: 0)	0.11%	0.62%	0.05%	0.12%	-	0.5%	0.60%	-	1.05%	-	-
Behenic acid (C 22: 0)	-	0.15%	0.01%	0.09%	4.3%	-	-	-	17.88%	-	-
Lignoceric acid (C 24: 0)	-	-	0.04%	0.09%	3.9%	-	-	-	-	-	-
Saturated	28.3%	36.3%	86.42%	22.2%	19.77%	14.3%	18.94%	32.28%	38.47%	7.70%	29.28%
Unsaturated	68.1%	63.7%	13.58%	77.8%	80.23%	85.7%	81.07%	67.71%	61.54%	95.2%	68.77%
Reference	[83,84,85]	[86,87,88]	[75,89]	[76]	[90,91]	[77,92]	[1,93,94]	[79]	[95]	[96]	[81]

**Table 3:** Minority compounds of different Amazonian vegetable oils by alphabetical order; (-) not reported.

Traditional Name	Buriti	Inchi	Patawa	Pequi	Pracaxi	Sacha inchi	Tucuma
Carotenoids	-	-	-	89.82 mg/kg	-	-	16.37 mg/kg
α-Carotene	76.8 mg/kg	-	-	-	-	-	-
β-Carotene	8.8 mg/kg	-	2.38 mg/kg	-	8.84 mg/kg	-	-
γ-Carotene	4.5 mg/kg	-	-	-	-	-	-
Aocarotenoids	0.5 mg/kg	-	-	-	-	-	-
Total carotenoids	1800 mg/kg	-	-	-	-	-	-
Squealene		11.7 mg/kg	-	-	-	-	-
Cholesterol	-	0.8 mg/kg	3.4 mg/kg	-	-	-	3.0 mg/kg
Δ5-Avenasterol	-	3.3 mg/kg	27.8 mg/kg	-	-	-	27.8 mg/kg
Cycloartenol	-	1.3 mg/kg	105 mg/kg	-	-	-	86.0 mg/kg
Methylenecicloartenol	-	-	-	-	-	-	-
Citrostadienol	-	0.8 mg/kg	-	-	-	-	-
Lanosterol	-	1.2 mg/kg	-	-	-	-	-
Campestanol	-	-	6 mg/kg	-	-	-	-
Campesterol	-	12.2 mg/kg	7.2 mg/kg	42.82 mg/kg	-	15.3 mg/kg	16 mg/kg
Stigmasterol	-	11.0 mg/kg	19.2 mg/kg	527.30 mg/kg	-	34.61–58.7 mg/kg	3 mg/kg
β-Sitosterol	-	55.0 mg/kg	34.2 mg/kg	238.50 mg/kg	-	43.46–127.4 mg/kg	61 mg/kg
α-Tocopherol	614 mg/kg	175 mg/kg	1.704 mg/kg	91.49 mg/kg	-	0.08 mg/kg	96 mg/kg
β-Tocopherol	687 mg/kg	9 mg/kg	-	-	72.92 mg/kg	0.02 mg/kg	2 mg/kg
γ-Tocopherol	50 mg/kg	575 mg/kg	-	63.82 mg/kg	416.13 mg/kg	127.6–149.0 mg/kg	1.8 mg/kg
δ-Tocopherol	136 mg/kg	57 mg/kg	-	-	7.78 mg/kg	60.0–84.0 mg/kg	-
Total tocopherol	1517 mg/kg	816 mg/kg	-	155.31 mg/kg	-	209–211.8 mg/kg	-
α-Tocotrienol	-	-	-	-	93.53 mg/kg	-	-
γ-Tocotrienol	12 mg/kg	-	269 mg/kg	-	-	-	55–59 mg/kg
δ-Tocotrienol	18 mg/kg	-	-	-	-	-	-
Referencias	[76,93]	[77,92]	[1,93,94]	[79]	[95]	[96]	[81]

Acai oil is rich in monounsaturated and polyunsaturated fatty acids, respectively, 60% and 14% of its composition. The main fatty acids present are oleic acid with an average concentration of 68.2%, followed by 17.5% palmitic acid. Regarding polyunsaturated fatty acids, linolenic acid and linoleic acid were the main compounds (Table 2) [97,98,99], while the lipid fraction acai comprises approximately 71% unsaturated fatty acids, with 60.8% monounsaturated and 10.14% polyunsaturated [83,84].

Andiroba oil is rich in fatty acids such as oleic, palmitic, stearic, and linoleic acids, together with 2% or 5% of the unsaponifiable material [74,100,101,102]. Of these compounds, limonoids, triterpenes, steroids, coumarins, flavonoids, and diglycerides have been isolated [103]. The limonoids, minority components isolated from andiroba, were 17β-hydroxyazadiradione, methyl angolensate, 7-deacetoxy-7-oxogedunin, deacetylgedunin, 6α-acetoxygedunin, gedunin, and andirobin [87,88].

Babassu oil is rich in lauric acid, myristic acid, and oleic acid (Table 2) [89]. Lauric fats are very important in industry, as they are resistant to non-enzymatic oxidation and, unlike other saturated fats, they have a low and well-defined melting temperature [104]. This oil has saturated and unsaturated fatty acids (Table 2), with 11.43% and 2.15% monounsaturated and polyunsaturated fats, respectively [75].

Buriti oil is rich in unsaturated fatty acids (oleic acid, linoleic acid, and linolenic acid) (Table 2) [76,105], characterized by high concentrations of nutraceutical compounds, mainly tocopherols, distributed in α-tocopherol, β-tocopherol, γ-tocopherol, γ-tocotrienol, ∂ tocopherol, and ∂ tocotrienol, and carotenoids, distributed in β-carotene, α-carotene and γ-carotene) (Table 3) [15,105,106,107,108].

Copaiba oil is an oil resin that consists of acid resins and volatile compounds [109,110,111]. The extracted oil can vary in relation to the concentration and nature of the diterpenes and sesquiterpenes present. The main sesquiterpenes found in the oil-resin of copaiba are β-caryophyllene, β-bisabolene, α-humulene, β-selinene, α-bisabolol, α-elemene, and γ-cadinene [5,112,113,114].

Cumaru seeds are characterized by having a high amount of unsaturated fatty acids, distributed in oleic acid, linoleic acid, and linolenic acid, and saturated fatty acids (Table 2) [73,90,91].

Inchi walnut oil has a high composition in terms of unsaturated fatty acids, distributed in linoleic acid as the main component, followed by oleic acid, linolenic acid, and palmitoleic acid (Table 2), while the saturated fatty acids are distributed in palmitic acid, followed by stearic acid, margaric acid, myristic acid, and arachidic acid (Table 2). Compounds of unsaponifiable hydrocarbons, sterols, and triterpenic alcohols are found (Table 3). Finally, among the isomers of vitamin E, there are alpha, beta, lambda, and delta tocopherol [77,92].

Patawa oil is rich in lipids, with 51.6% dry weight. Its high degree of unsaturated fatty acids is remarkable [68]. Patawa oil presents in steroids, β-sitosterol, Δ5-avenasterol, stigmasterol, campesterol, campestanol, and cholesterol. It also has carotenoids (β-carotene) and tocopherols (Table 3) [68,94].

Pequi oil has been widely explored due to its high content of seeds and pulp, which reaches around 70.16% w/w. The seed oil contains mainly palmitic acids and oleic, followed by linoleic acid and linolenic (Table 2). This oil also has a large amount of lipid antioxidants, mainly tocopherols, carotenoids, and phytosterols (Table 3).

Pracaxi oil is composed of various fatty acids, with oleic acid and behenic acid, followed by linoleic acid and linolenic acid (Table 2), constituting 96% of the total fatty acids present in this oil. Pracaxi oil contains the highest known concentration of behenic acid, six times higher than that found in peanut oil [73] and is often used in the cosmetic industry for application in makeup and hair products, due to its excellent moisturizing properties [95,115,116].

Sacha inchi oil contains lipids (35 to 60%), of which 97.2% are neutral lipids, with 9.65% corresponding to oleic acid, linoleic acid, and linolenic acid (Table 2) [96], 1.2% to free fatty acids, and 0.8% to phospholipids [117] proteins (25 to 30%) (including essential amino acids such as cysteine, tyrosine, threonine, and tryptophan), vitamin E, polyphenols, and phytosterols, among others [80,96,117,118,119,120,121]. Cosmetic and pharmaceutical preparations containing sacha inchi proteins and oils have been patented [patent number, US2007264221 (A1)].

Tucuma oil contains saturated fatty acids and unsaturated fatty acids, represented by palmitic, stearic, oleic, and linoleic fatty acids (Table 2) [81,82] and it is rich in omega 3, 6, and 9 [81]. Among the minor components present are tocopherol, total sterols, diacylglycerol and total carotenoids (Table 3) [121].

Therefore, these results provide information on alternative natural Amazonian oils that could be used as optional sources of raw material for the pharmaceutical and cosmetic industries. Many of these oils are potential solvent candidates in organogelation processes, opening a new opportunity for the synthesis of organogels as active transporters or even to convey some bioactive substances present in the oils. The physicochemical characteristics of these oils, both in the proportion of unsaturated fatty acids and in the presence of minor polar ingredients, could improve many of the properties in the formation of the three-dimensional network, as well as in its application, since many of these compounds provide a high degree of nutrition to the skin. It is worth mentioning that the organogel structure generates oxidative protection for the bioactive compounds in the oil or the pharmacological active ingredients involved in the formulation, as mentioned below.

The importance of traditional natural oils in the synthesis of organogels has been widely studied, mainly in the structuring or self-assembly of organogelators through non-covalent interactions, depending on the vegetal oil properties, such as the amount of unsaturations and polyunsaturations and the presence of TAG, DAG, and MAG, followed by polar minority compounds. Many of the characteristics of micro and macro structures are mediated by the organogeler and by the nature of the solvent, which in turn have an effect on ease of preparation, their ability to dissolve lipophilic agents, their thermo-reversibility, and their ability to control mechanical properties and texture. In this way, Amazonian oils show a wide spectrum of possibilities in the synthesis of organogels, thanks to their high concentration of unsaturated and polyunsaturated fatty acids, which greatly exceeds traditional natural oil and improves penetration through the lipid bilayer of the stratum corneum, being the main permeability enhancers [122,123,124,125]. In addition, polar minority compounds and unsaponifiable compounds give some of the characteristics to Amazonian oils and can affect the formation of the micro and macro structures system.

This is how a new chapter opens in the synthesis of organogels with non-traditional Amazonian solvents, which, until now, have been little explored in this type of transdermal development. Possible advantages and differentiating characteristics compared to organogels synthesized with traditional solvents are texture, rheological behavior, oxidative stability, ease of involving active ingredients, synergism due to the presence of minority polar and unsaponifiable components such as vitamins, and ease of transdermal penetration. Furthermore, the use of Amazonian solvents is promising for topical and transdermal administration systems, due to their low cytotoxicity and hypoallergenicity, thus offering a biocompatible and sustainable alternative.

## 6. Oxidative Stability of Organogels

The advantage of making this type of organogel does not only lie in the type of micro or nano structure that can be obtained and the purpose to which it can be directed, but also the protective effect that the three-dimensional network provides to the constituents of the oil, providing oxidative stability against environmental variables such as oxygen, light, and heat, among others [126,127]. Authors such as Hwang et al. [41] suggest that this protection is owed to the immobilization of the oil in the gel structure, which leads to a lesser loss of antioxidant compounds and unsaturated and polyunsaturated fatty acids, which are related in many cases to improving absorption of the skin and its nutrition content.

Some studies such as Silva et al. [127] used sunflower oil with a high amount of oleic acid. They conclude that the use of organogels generates thermal stability. In the work of Ferrer et al. [128], it was ensured that the organogel structure, using soybean oil, had a lower lipid oxidation value than the control, which indicates that the three-dimensional structure of the organogel generates oxidative stability in the oil. In the study of Tian [126], the photostability of the organogel network on retinyl palmitate was observed. It was suggested that policosanol blocks the energy absorption of UVA rays and dampens the photoirradiation of retinyl palmitate (ionic photodissociation and reaction of free radicals) mediated by UVA, due to the immobilization of the matrix, a result that agrees with the work of Cui et al. [129] and Zheng et al. [130], where it was established that the organogel improves the solubility and chemical stability of β-carotene against UV radiation with storage temperatures of 25 °C and 55 °C. However, in the work of Öğütcü et al. [131], there was no significant difference between gelled cod liver oil and ungelled cod liver oil.

This discrepancy in the results may be mainly due to the analytical method used for the analysis. Both studies used oil and synthesized organogels, which many authors related to the standard procedures dictated by AOAC (Association of Official Agricultural Chemists), AOCS (American Oil Chemistry Society), and IDF (International Dairy Federation), among others. This oxidative stability must be measured by a standardized analytical method in which all the physicochemical characteristics of the oil, the active ingredient, and the organogel are involved.

In addition, the concomitant detection of primary and secondary oxidation products of the oil and the active ingredients must be performed in order to make a correct comparison or, failing that, multiple reliable analytical methodologies must be provided to test the numerous products obtained from oil oxidation. A single analytical method is known to detect only one type of oxidation product at a time [132,133,134]. The type of organogelator used can also cause discrepancies in the results. As previously mentioned, organogelator of natural origin, such as waxes, have a large amount of compounds that can affect the gelling processes, the texture of the organogels, and their oxidative stability, among other characteristics [134]. In addition, some of the ingredients of the waxes are pro-oxidants [36,41,132].

## 7. The Skin

The skin is an organ of great multifunctional importance. It promotes, through the skin barrier function, the protection against dehydration, generates an immunological surveillance against aggressive microorganisms, and has a thermoregulatory effect [135,136,137]. Therefore, it is more or less permeable to chemicals. It allows the passage of drugs under certain conditions [137]. There are several topical pharmaceutical forms that are absorbed.

The skin consists of three layers. The epidermis, the outer layer of the skin, is subdivided into five layers or strata, which are (from the outer layer) the germinative stratum or basal stratum, stratum spinosum, stratum granulosum, stratum lucidum, and stratum corneum [137,138,139]. The dermis is the connective tissue on which the epidermis rests and joins the skin to the subcutaneous cellular tissue or hypodermis [140]. It is characterized as acellular, but it is rich in blood vessels, lymphatic vessels, and nerve endings [140,141]. The hypodermis already functions as a mechanical shock absorber and thermal barrier, rapidly synthesizing and replenishing readily available energy substances [140].

From the technological viewpoint of drug delivery systems, the skin is considered a gateway to therapeutic agents [142]. This possibility encourages the development of formulations capable of transposing the barrier imposed by the skin and delivering drugs to the site of action without causing damage to its integrity and the main physiological function of protection [143]. Knowing the parameters that influence the permeability of the skin is important to be successful in topical therapy. The use of pharmaceutical forms for topical application has as its objectives a local effect with cosmetic action, drug transport through the skin seeking a systemic effect, superficial action with the absence or reduction of a systemic effect, reaching deep layers of the skin, and, finally, not absorbing in any layer of the skin [144,145]. The routes of penetration into the skin are diffusion through the epidermis through the attached structures of the skin (hair follicles and glands), intercellular (interlamellar regions in the stratum corneum), and intracellular. Each one presents the necessary characteristics for the type of effect desired [141,146].

The study of the penetration and cutaneous permeation of active ingredients through the skin may present the epidermis as the first limiting factor, especially the stratum corneum, due to its barrier function and because it is the first layer of contact with the external environment [147,148]. A recognized effective strategy to favor the absorption of substances through the skin is the use of penetration promoters; however, the incorporation in cosmetic or pharmaceutical vehicles is restricted due to the scarce information of their commonly complex mechanisms of action [148,149].

### 7.1. Skin Permeation

For percutaneous absorption, it is necessary for the substance to penetrate the skin through the outermost layer, the stratum corneum. It is made up of keratinocytes and a lipid matrix [149,150]. Keratinocytes are dead cells that consist of keratin embedded in a lipid matrix. The lipid matrix is organized in lamellar bilayers, which consist mainly of triacylglycerols, free fatty acids, ceramides, and sterols [138,149,151]. Therefore, the difficulty of the drug to penetrate the stratum corneum (organized structure) is due to lipid bilayers and keratinocytes [149,152]. The active ingredient can penetrate the skin, crossing the stratum corneum through two different pathways (transcellular, through the corneocytes (keratinocytes) and the lipid matrix, or through the intercellular pathway, between the corneocytes and the lipid matrix) or even crossing the skin through the skin tags [142,149].

**Figure 3:**
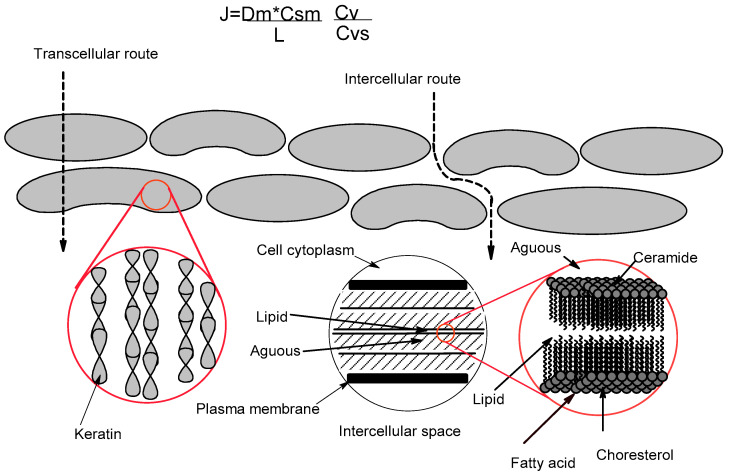
Permeation routes through the stratum corneum: via the lipid matrix between the corneocytes (intercellular route) and across the corneocytes and the intercellular lipid matrix (transcellular rout) [146].

The permeation of the active ingredients through the skin is described by means of Fick’s first law (Figure 3), where J is the drug flux through the stratum corneum, Dm is the diffusion coefficient of the active ingredient, Csm is the solubility of the active, L is the thickness of the membrane (skin), cv is the concentration of the active dissolved in the vehicle, and Csv is the solubility of the active in the vehicle [146].

The absorption of the active through the skin depends on the chemical nature of the compound. The substance must have a molecular weight lower than 0.6 KDa, partition coefficient of 1 to 3 (adequate solubility in water and oil), and not be in ionized form [153]. Few actives have these characteristics and, therefore, cannot penetrate the skin in large quantities if they are not incorporated in appropriate topical formulations [139,150,154].

The increase in the diffusion coefficient of the active through the skin can be obtained by promoting the disorganization of the lipids that constitute the stratum corneum. This disorganization allows a greater diffusion through the stratum corneum [155,156]. The most common transdermal vehicle is Pluronic Lecithin Organogel (PLO) [32,157]. PLO modulates the release and permeability of active ingredients applied transdermally [32,157]. Lecithin can act by increasing the fluidity of the stratum corneum as it disorganizes the structure of the skin, temporarily opens the pores of the skin, and increases the penetration of active ingredients [155,158]. However, the exact mechanism of how lecithin alters the skin is not yet clearly understood [141,156,158]. Lecithin-containing gels have been shown in vitro to increase transdermal penetration of many different agents [159].

Fatty acids are other substances that disorganize the lipids of the stratum corneum. They are often used for this purpose, and oleic acid is an example, as it induces the separation of the lamellar phases of the lipids of the stratum corneum [150,152,160,161]. Oleic acid markedly increased the permeation coefficient of melatonin through hairless rat skin by more than 950 times compared to that obtained with propylene glycol used as a vehicle alone. Therefore, oleic acid used in a suitable vehicle has been shown to be a more effective promoter of melatonin permeation than with ethanol, polyethylene glycol, propylene glycol, and their binary mixtures [139].

### 7.2. Organogel Tecnology as a Controlled Release Mechanism

Organogel technology is an innovative method of structuring organic fluids and has aroused great deal of interest due to the potential to structure vegetable oils with small concentrations of organogelator, also of plant origin. This technology is based on principles that promote sustainability, as it is highly economical and uses natural, renewable, and rapidly degrading products. Its potential in the cosmetic area ranges from the simple structuring of a vegetable oil as a substitute for petroleum jelly to acting as a technological vehicle, with the capacity to load assets capable of offering greater permeation to the skin [162]. In 1990, PLO (Pluronic^®^ lecithin organogel) was developed as a vehicle for topical and transdermal drug delivery by Marty Jones and Lawson Kloesel in an American pharmacy [163]. They prepared the original lecithin organogel (LO) by adding an amount of water to an organic lecithin solution and added the aqueous phase of the formulation to the polymer Polaxamer F127 [154,155,164].

Since then, lecithin organogels have been widely explored due to their composition of phospholipids that are biocompatible with the skin, which can facilitate the permeation of assets. An increase in viscosity was also noted as a secondary benefit of the technique [61]. The rheological characteristics of pluronic organogels, a transdermal excipient widely used in the pharmaceutical industry, and the release of lecithin anti-cellulite active ingredients were comparable to those of pluronic hydrogels. Hydrogels, due to their great capacity to swell in the presence of aqueous solvents, form a three-dimensional gel network. In association with organogels, they can retain hydrophobic and hydrophilic agents, which are released from the gel mesh for the skin through manipulation of the diffusion and permeation characteristics [165].

Another line of research for the formation of organogels is the modification of guanosine. Guanosine (G) is a type of important nucleoside originated from RNA. It can form different self-assembled species through intermolecular H bonds, including G-dimer, G-ribbon, G-quartet, and G-quadruplex [166]. A particularly interesting application in G-quadruplexes is the formation of gels for tissue engineering, cell culture, and drug delivery [167]. Compared to G-hydrogels, G-organogels are less studied, possibly due to limited functional sites to meet gelation and solubility requirements [168]. In general, lipophilic modification in ribose is often necessary to provide additional hydrophobic interaction to stabilize the fibrous network. In the previously reported G-gel systems, ribose modification is the dominant approach to building a fibrous network. The guanine part serves only as an H link conveyor. The only variable synthetic cable in guanine, position C-8, remained less developed for gelation. Gel formation using modified C-8 guanosine derivatives has been reported; substituents at position C-8 affected the conformation of guanosine (syn- and anti-ratio) and further influenced the property of the gel [169,170].

In cross-linked oil systems, drugs are incorporated into the oil or water phase depending on their physical and chemical characteristics. In addition, some solvents such as propylene glycol can be used to deliver lipophilic drugs before dispersing them in the oil phase. Some drugs that are incorporated into the PLO (Pluronic lecithin organogel) are hormones, such as estriol and estradiol; non-steroidal anti-inflammatories, such as ketoprofen, piroxicam, and diethylammonium diclofenac; serotonin reuptake inhibitors, such as fluoxetine and paroxetine; cyclobenzaprine with lidocaine; and dexamethasone [29,153,154,171]. The cross-linked oil systems have a gel mesh microstructure capable of acting as a matrix to trap lipophilic assets, as is the case with photoprotective dermatological assets, which Kirilov et al. [172] studied the efficiency of for systems containing organogel nanoparticles dispersed in water in the presence of photoprotective molecules. The use of these systems was related to the increased efficiency of waterproofing, as well as to a greater photoprotection and photostability of the filters. Oil structuring technology was explored in the nanotechnology area with the aim of using cross-linked oil systems to share the structure of nanoparticles or to act as a matrix in its release [156,168,173].

Organogels can be classified based on the properties of gelling agents, solvents, and intermolecular interactions that turn into gels. The ease with which these gels penetrate the skin was debated several years ago. Many researchers, such as Willimann et al. [174], reported that the lecithin organogel interacted with the stratum corneum of the skin and disorganized the lipid layers so that the active ingredient it carried penetrated more easily. It is possible that the proposed disorganization was due to the interaction between skin lipids and phospholipids used in the preparation of the cross-linked oil systems.

Despite the potential, the technology is recent and its application is still very timid. However, innovative products are found on the market in which organogel technology has been successfully applied. Recently, in the beauty industry, a permanent hair color without ammonia was released, which uses this technology as a vehicle for the penetration of pigment through the hair cuticle. In the development of this product, the knowledge about the cross-linked oil system in the pharmaceutical area successfully migrated to the cosmetic area [175]. Additionally, it is during the development phase that many of the in vitro and in vivo studies are necessary to evaluate the ability of this organogel to act as a vehicle for site-specific active ingredients.

During the development phase of dermatological products, it is through in vitro studies of skin release and absorption that it is possible to select the most suitable excipients for the dermal release pharmaceutical dosage forms, in order to provide adequate release of active ingredients, thus contributing to the achievement of the desired therapeutic effect [176,177,178]. The ability of a drug, present in topical formulations, to permeate the skin depends on its ability to release the vehicle to the skin and to allow diffusion through this barrier to its site of action [176,177,178].

In vitro skin permeation models used to evaluate topically applied drugs use vertical diffusion devices. The Franz diffusion cell is the most widely used model of this type [160,179,180]. The Franz cell has a donor compartment and a receptor compartment separated by a membrane, which can be biological or synthetic (cellulose acetate, for example) [160,179,180]. The receiving compartment must provide sink conditions for constant diffusion of the drug from the donor environment to the recipient environment. For hydrophilic compounds, the receptor medium composed of a buffer solution at physiological pH is sufficient to meet the sink conditions; however, for lipophilic drugs, it is necessary, in some cases, to use additives that promote greater solubility of the same in the receptor environment [151,153,161]. The temperature of the cell is controlled by a thermal water bath in order to maintain the temperature close to the physiological temperature of the skin [179,180]. In these systems, skin or synthetic membranes can be used as a barrier to drug flow and a vehicle to simulate an in vivo system [179,180].

The biological membrane consisting of human skin tissue is considered the gold standard for this type of in vitro assay; nevertheless, this shows great viability between samples for differences between race, sex, age, and anatomical site of donor tissue removal [123,124]. Furthermore, its use involves issues that require the approval of an ethics committee to carry out experiments with this type of human tissue [123,124]. In this sense, substitutes for human skin have been sought, such as the skin of pigs, guinea pigs, and mice, although they have higher permeability compared to human tissue, as well as the use of snakeskin [123,124].

Pig tissue is widely used because it can be easily obtained from animals that have been slaughtered for human consumption and because it has morphological characteristics similar to human skin tissue [181]. Comparative studies indicated that the in vitro permeation of water and chemicals, such as drugs, for example, was similar between pig and human tissues under the same experimental conditions [123,181]. Pig skin has a hair distribution (20 hairs per cm 2), stratum corneum thickness (21–26 μm), epidermis (66–71 μm), dermis, and elastic tissue content similar to that of human skin tissue [123]. The major differences between the skin of humans and pigs are vascularization and the proportion of sebaceous glands [181]. Vascularization is lower in porcine tissue and the proportion of eccrine glands is higher in human tissue, while in pigs only apocrine glands are present [181]. In these cases, guinea pig skin can also be used as an alternative membrane in in vitro studies of skin penetration and/or permeation tests because it also has a correlation with human skin. Snake skin also tends to resemble the stratum corneum of human tissue, although it does not contain hair follicles, and can also be used in such studies [123,124,181].

The quantification of the drug present in the skin as a whole or in layers was carried out using several techniques. The technique used to quantify the drug present in the stratum corneum is known as tape stripping, which consists of the progressive elimination of the stratum corneum with the use of adhesive tapes [124,152,160]. The application of the tape to the skin and subsequent abrupt removal results in a layer of the stratum corneum and the drug that possibly penetrated this layer adhering to the adhesive tape. Several removals were performed to achieve complete removal of the stratum corneum from the skin [124,152,160]. The accumulated amount permeated is related to time, and the permeation in the equilibrium state J (Equation (1)) is calculated from the slope of the line of the linear part of the permeation profile obtained. The intersection of the line that refers to the linear part of the permeation profile on the time-related axis corresponds to the delay time, which is the time necessary for the onset of permeation to occur [123,124,152].

Another widely used technique to quantify the permeation of the active ingredient in the skin is the Franz diffusion cells, which have a donor compartment, where the sample is placed, which comes into contact with the skin and is, in turn, in contact with the receptor compartment. This Receptor medium is generally a phosphate buffer solution at a pH of about 7. Zhang et al. [182] evaluated the transdermal permeation of drugs modulated by Lipoderma and Pluronic lecithin organogel (PLO) on porcine tissue using Franz cells. The results showed that the hydrophilic compound significantly increased penetration and retention in the skin. Simsolo et al. [183] Formulated and optimized microparticles in a caffeine-loaded organogel system for the synergistic effect of long-term treatment of cellulite using diffusion cells of the Franz type through the abdominal skin of a Wistar Albino rat.

Kirilov et al. [24] investigated the percutaneous absorption of enrofloxacin from two formulations of organogel obtained from isopropyl myristate and oleic acid as an oily phase and 12-hydroxystearic acid as LMOG through a pig ear skin model. Ba et al. [184] performed the permeation study using rats’ skin in Franz diffusion cells in vitro and in vivo microdialysis from the sinomenine puronic lecithin organogel system and recommended that PLO can be used as an advantageous transdermal delivery vehicle to increase sinomenine permeation and deposition in the skin. These examples show us that Franz cells have a wide spectrum of uses both in the evaluation of permeability depending on the tissue to be evaluated and in the active ingredients used and their lipophilic and hydrophilic characteristics.

## 8. Conclusions

The understanding of the relationships between composition, microstructure, and physicochemical properties of Amazonian oils in the synthesis of organogels is still very limited. The contribution of the chemical structure, especially of polyunsaturated fatty acids and minority components, in the properties of cross-linked oil systems is still a field to be explored. Information on critical quantities of structural agents is needed for the formation of the three-dimensional network, as well as synthesis conditions aimed at preventing nutraceutical degradation of the Amazonian oils. The relationship between the composition of the oil and the macrostructure formed from the organogelator is important to study, as well as the mixing behaviors of multicomponent systems, as in the case of lecithin organogels. The effects of molecular interactions and mixing ratios on mechanical strength, thermal properties, and appearance of synthesized, cross-linked oil systems are also relevant concepts to be investigated.

This review presents a general description of the influence and importance of the selection of gelling agents and natural solvents in the synthesis of organogels with cosmetic application, highlighting the importance of Amazonian oils and their exceptional characteristics. Some of these favorable properties include their composition of saponifiable substances such as mono- and polyunsaturated fatty acids, which provide deeper penetration into the skin, as well as unsaponifiable substances that nourish the skin (terpenoids, phytosterols, eicosanoids, and fat-soluble vitamins, among others). Many of these oils are potential solvents in the synthesis of organogels, opening new avenues of exploration of macrostructures and matrices as active ingredient transporters or even to transport some bioactive components of the Amazonian oils themselves.

The synthesis of these systems using Amazonian oils has shown us that both the melting point and solubility of the gelling agent in the natural solvent considerably affect the characteristics of the organogel. In the same way, it was established that the minority components of Amazonian natural solvents could have a drastic effect on both the macrostructure and its physicochemical characteristics. However, in-depth studies are still required on the mechanisms that these minority compounds have on the gelling process.

It was also possible to establish the importance of the entire Amazon in the potential development of organogels, as they are easy-to-manufacture, safe, and efficient systems, since many of these oils are currently exploited mainly by the food industry and the cosmetic industry, always with a policy for the sustainable management of natural resources.

## Data Availability

Data are contained within the article.
